# Reliability of Monothermal Caloric Test as Screening Test of Vestibular System

**DOI:** 10.3390/jcm11236977

**Published:** 2022-11-26

**Authors:** Salman F. Alhabib, Issam Saliba

**Affiliations:** 1 Department of Otolaryngology-Head and Neck Surgery, King Abdullah Ear Specialist Center, College of Medicine, King Saud University, Riyadh 11451, Saudi Arabia; 2 Department of Otolaryngology-Head and Neck Surgery, Montreal University Hospital Center (CHUM), Montreal University, Montreal, QC H2X 3E4, Canada

**Keywords:** caloric test, vestibular test, bithermal, monothermal, vestibular paresis, caloric paresis, vertigo

## Abstract

This retrospective study completed at a tertiary care center aimed to assess the monothermal caloric test (MCT) as a screening test, using the bithermal caloric test (BCT) as a reference. Additionally, it attempts to measure the sensitivity, specificity, positive predictive value (PPV), and negative predictive value (NPV) of a fixed inter-auricular difference (IAD) value for both cold and warm stimuli using water irrigation. Medical records of 259 patients referred for vestibular symptoms who underwent BCT with water irrigation were reviewed. Patients with bilateral vestibular weakness and caloric tests using air irrigation were excluded. BCT showed 40.9% unilateral weakness. Two formulas were used to determine the monothermal caloric asymmetry (MCA-1 and MCA-2). The measurement of agreement Kappa between the two formulas in comparison with BCT revealed moderate agreement at 0.54 and 0.53 for hot and cold stimulation, respectively. The monothermal warm stimulating test (MWST) using MCA-2 showed better results, with a sensitivity of 80%, specificity of 91%, PPV of 83.1%, and NPV of 89.2%. Thirty-four patients had horizontal spontaneous nystagmus (HSN) with a mean velocity of 2.25°/s. These patients showed better sensitivity but lower specificity after adjustment of HSN using the MCA-2 formula at warm temperatures. Therefore, they should complete the caloric test with cold irrigation to perform the BCT. MCT is efficient as a screening test if the warm stimulus is used with the MCA-2 formula fixed at 25%. If present, HSNs should be adjusted. Negative IAD (normal) in the absence or presence of adjusted HSN or slow-phase eye velocity ≤ 6°/s at each right and left warm stimulation should be accomplished by the BCT.

## 1. Introduction

The bithermal caloric test (BCT) assesses the function of the lateral semicircular canal by generating thermal variations within the external auditory canal. This variation in temperature changes the density of the endolymph fluid within the lateral semicircular canal and leads to a convection current that stimulates sensorial cells located in the lateral ampullary crest.

Thermal variation in the standard BCT is achieved by four irrigations (cold and warm stimulation for the right and left ear) using water or air stimulation that are warmer or cooler than the body temperature. The slow-phase eye velocity (SPEV) of nystagmus is generated by stimulation of the lateral semicircular canal with water irrigation. Then, the unilateral weakness (UW) and direction preponderance (DP) are calculated using the Jongkees formulas [1]. Caloric tests and video head impulse tests are the gold standard tests for evaluating the function of the vestibular system in vertiginous patients [2,3]. However, the caloric test is the most uncomfortable vestibular test because it can induce nausea and vomiting, and some patients refuse to complete the test. Furthermore, the BCT is time-consuming, taking at least 40 min depending on the operator, patient compliance, and laboratory protocol.

The monothermal caloric test (MCT) assesses the function of each lateral semicircular canal by using single-temperature irrigation. Compared to BCT, MCT is more economical, less time-consuming, and offers less patient discomfort during the test. To be considered a screening test, MCT must have high sensitivity and acceptable specificity. Monothermal caloric asymmetry (MCA) was calculated by many authors using the following formula: MCA = (right ear − left ear) × 100/larger stimulated side [4]; another formula is also used: MCA = (right ear − left ear) × 100/(right ear + left ear) [5] where the peak SPEV of the response following the temperature irrigation is reported for each side.

Barber et al. investigated the efficiency of a monothermal warm screening test (MWST) in 1971 [6]. He used the results of BCT on normal subjects as the gold standard. He found a false negative result of MWST equal to 0.7% if the inter-auricular difference (IAD) was less than 23% and each of the irrigations induced a caloric nystagmus with a SPEV greater or equal to 11°/s (SPEV ≥ 11°/s) (1). Many authors have proven that MCT is an invalid screening test because of the high false negative predictive value reaching 29% [7,8,9] and high false positive predictive value ranging from 48% to 78% [9,10]. On the other hand, many authors found that MCT is a valid test with warm water irrigation after excluding the failure criteria [11,12] that could increase the false negative rate; therefore, it needs to be completed by a BCT; these failure criteria are: (1) IAD lower than 15%; (2) SPEV less than 8°/s for each warm irrigation [13]; (3) horizontal spontaneous nystagmus (HSN) of 4°/s or higher [5,12,13]. Different study group sizes, different failure criteria for pathologic BCT, variations in test procedures, and stimulus types will result in conflicting results and different conclusions. The aims of this study were (1) to confirm the reliability of the MCT as a screening test using the BCT procedure as a reference in patients with and without HSN, and (2) to assess the correlation between the two formulas used to measure the MCA in comparison to the BCT results. In addition, we measured the sensitivity, specificity, positive predictive value (PPV), and negative predictive value (NPV) in all patients with the IAD value for both warm and cold stimuli using water irrigation.

## 2. Materials and Methods

This retrospective study was conducted at our tertiary care center for a one-year duration. This study was approved by the institutional review board of our institution. The BCT is a standard screening test used to evaluate the function of the vestibular system. We reviewed the medical records of all patients with signs and symptoms of vestibular system dysfunction. Bilateral vestibular weakness and caloric tests using air irrigation were excluded from the study to decrease bias. Bilateral vestibular weakness was defined as a peak SPEV lower than 6°/s at each of the four caloric irrigations [14]. Patients with HSN will be included and studied separately to avoid bias in the results and conclusions.

A caloric test using water irrigation was used in combination with videonystagmography (VNG) to test patients with dizziness and vertigo. The VNG evaluates the oculomotor system, which includes the saccadic, gaze, optokinetic, and pursuit systems. It also includes spontaneous, positional nystagmus, and the caloric test. Caloric stimulation was performed using an open-loop water irrigation unit. Water irrigation was performed with subjects in the supine position and heads in flexion at 30°, using warm 44 °C and cold 30 °C in each ear for 20 s for a total volume of 250 mL. In all patients, an alternating ear sequence was used, starting from the right ear in the following order: right warm, left warm, right cold, and left cold. A minimum interval of 5 min was allowed between the caloric subtests.

Bithermal caloric asymmetry (BCA) was calculated using the Jongkees formula: BCA = [(Right Cold + Right warm) − (left cold + left warm)] × 100/(right cold + right warm + left cold + left warm), where the peak SPEV of the response following temperature irrigation is reported for each side [15].

MCA for both warm and cold conditions was calculated as MCA-1 = (right − left) × 100/larger stimulated side [4] or MCA-2 = (Right − Left) × 100/(Right + Left) [5].

The unilateral vestibular weakness (UVW) is defined in BCA, MCA-1, and MCA-2 as IAD ≥ 20% [14], ≥25% [10], and ≥25% [3], respectively. We fixed the cutoff point of UVW for these values based on the reported references.

### 2.1. Horizontal Spontaneous Nystagmus Adjustment

It is expected that a patient with an acute unilateral peripheral lesion will have spontaneous nystagmus with a constant slow-phase velocity with their eyes closed. Spontaneous nystagmus skews the results of the caloric test and must be accounted for by adding (or subtracting) the numeric value of spontaneous nystagmus from each caloric subtest (note that some software systems are automatically corrected for spontaneous nystagmus). Spontaneous nystagmus is almost always “direction-fixed” and will therefore “add” to the responses opposite to the direction of the spontaneous nystagmus fast phase and “reduce” the responses in the same direction of the fast phase. For example, a 4-degree right-beating spontaneous nystagmus adds 4°/s to the value of both the right warm and left cold responses. Therefore, for adjustment, 4°/s should be subtracted from each of the peak SPEV values to not report a direction proportional to what was actually due to spontaneous nystagmus. Likewise, for the right cold and left warm responses, the effect would be the opposite (i.e., 4°/s would be added to each subtest to correct for spontaneous nystagmus).

### 2.2. Statistical Analysis

Kappa was used to define the agreement between the MCA-1 and MCA-2 formulas with the Jongkees formula of BCT. Then, the measurement of agreement Kappa was used to study the agreement between the BCT and MCT (MWST and MCST: monothermal cold stimulation test). Pearson’s product-moment coefficients were used to examine the correlation between the BCT and MCT. The area under the ROC curve was calculated to show the correlation between the sensitivity and specificity of the MWST and the MCST. ROC curves displaced into the upper left corner of each panel indicate an excellent screening test with high sensitivity and specificity. We also measured PPV and NPV. All analyses were performed using SPSS version 24. *p* value greater than 0.05 is considered statistically significant.

## 3. Results

**Figure 1:**
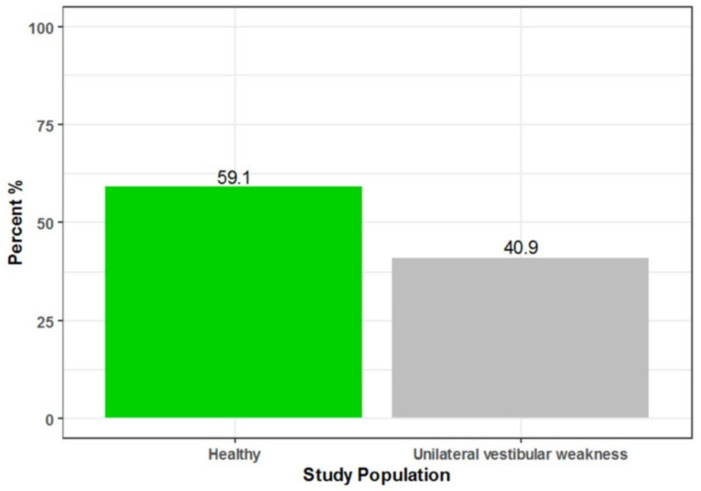
Patient with unilateral vestibular weakness and healthy controls.

A total of 259 patients with VNG studies were included; 60.1% were female and 39.1% were male; the median age was 50.4 years;

The patients included in this study were presented to the neurotology clinic to rule out the causes of vestibular dysfunction. The study group showed 40.9% UVW and 59.1% without vestibular asymmetry (healthy controls), as shown in Figure 1. The female sex was the majority in both groups (Table 1).

When the formulas MCA-1 and MCA-2 were fixed at 25% and BCA was fixed at 20%, the measurement of Kappa agreement for the inter-rater reliability between the three screening tests was 0.54 for hot stimulation and 0.53 for cold stimulation, indicating a moderate agreement between the three screening techniques. Table 2 shows the results of both the MWST and MCST in comparison to the BCT results as a reference gold standard using both the MCA-1 and MCA2 formulas among 225 patients without HSN. The MCA-2 formula showed higher sensitivity (80%) and specificity (91%) in the MWST, with high positive and negative predictive values of 83.1% and 89.2%, respectively. At both temperatures, the MCA-1 formula showed better sensitivity than the MCA-2 formula, but with lower specificity. Furthermore, the MCA-1 formula showed a lower PPV in both the MWST and MCST than the MCA-2 formula. The NPV was high for both formulas and both the MWST and MCST, as shown in Table 2. The false PPV and false NPV for the MCT were lowered by using MCA-2 in hot stimulation and were 16.9% and 10.8%, respectively.

**Figure 2:**
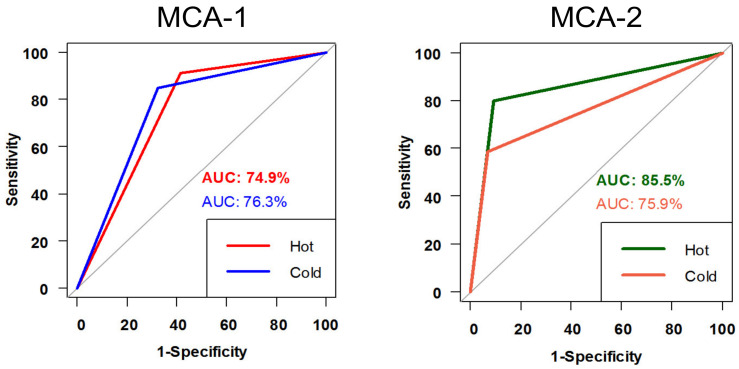
ROC curve for MCT for both warm & cold stimulation among non-HSN patients by using the two formulas: MCA-1 and MCA-2. (ROC curve: receiver operating characteristic curve, MCT: monothermal caloric test, HSN: horizontal spontaneous nystagmus).

The ROC curve results for the sensitivity and specificity of the MCT for both warm and cold conditions in patients without HSN were better using the MCA-2 formula. The MWST performed better under the curve (85.5% using the MCA-2 formula) than the MCST (75.9%) (Figure 2). The MWST and MCST in the MCA-1 ROC curve results of patients without HSN were 74.9% and 76.3%, respectively. The *p*-values for the ROC curve for both the MCA-1 and MCA-2 formulas were significant (*p* < 0.001) at hot and cold temperatures.

A total of 34 patients had HSN with a mean velocity of 2.25°/s (2.25 ± 1.6°/s). Sensitivity, specificity, PPV, and NPV with and without adjustment of the HSN using the MCA-2 formula to measure the MCA showed better results than the MCA-1 formula (shown in Table 3). The sensitivity was better after the adjustment of the HSN for both cold and warm stimulation. The specificity was better only in cold stimulation with an adjusted HSN. With HSN adjustment, NPV was higher for both cold and warm stimulation. As the cold stimulation showed better results with HSN adjusted, the false PPV and false NPV in cold stimulation with HSN adjusted were 20% and 35.7%, respectively.

**Figure 3:**
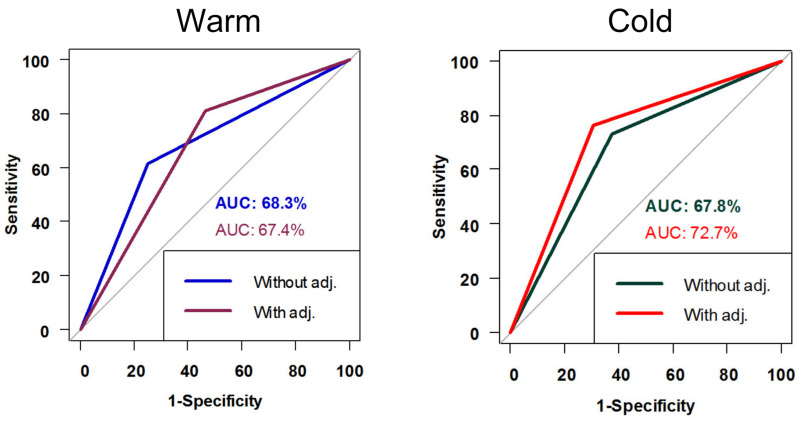
ROC curve for MCA-2 with and without HSN adjustment for both warm and cold stimulation.

The ROC curve results for the sensitivity and specificity of MCT in patients with HSN, with and without adjustment for both warm and cold stimulation, are shown in Figure 3. The results for both the formula and both warm and cold stimulations were better after the adjustment of HSN. The MCA-2 with cold stimulation showed the best result under the curve after adjustment of the HSN, with a result of 72.7%. The *p*-value for the ROC curve for MCA-2 using cold stimulation before adjustment for HSN was 0.037 and became more significant after adjustment for HSN at 0.005. The *p*-value for the ROC curve for MCA-2 using hot stimulation before the adjustment of the HSN was 0.039 and became more significant after the adjustment of the HSN at 0.02.

## 4. Discussion

This study aimed to confirm the reliability of MCT in differentiating healthy individuals from individuals with UVW. Our study group showed that 40.9% have a unilateral right or left vestibular weakness. MCT has an advantage over BCT as it offers less discomfort to the patient, is less time-consuming, and is more economical. On the other hand, it has to be as reliable as a BCT, with high sensitivity and acceptable specificity for use as a screening test in specialized neurotology clinics. The DP results of BCT were not included in the study, as it requires four irrigations to be calculated, it does not represent asymmetry, such as UW, and it could deviate to either side without the presence of HSN [10,11,12,13,15,16]. Previous studies using water caloric tests have shown that the sensitivity of the MWST is higher than that of the MCST, and the specificity is nearly similar [5,7,8,11,12,17,18]. In contrast, Enticott [10] and Cunha et al. [16] found the sensitivity and specificity of the MCST to be more reliable than those of the MWST. Our study showed that the sensitivity, specificity, PPV, and NPV were higher in the MWST (80%, 91%, 83.1%, and 89.2%, respectively) than in the MCST (58.8%, 93.1%, 82.5%, and 80.4%, respectively) using the MCA-2 formula and in patients without HSN. This thermal variation could be explained by the fact that warm irrigation will produce an action potential due to the stimulation of the sensorineural cells in the crista ampullaris of the lateral canal, generating depolarization, whereas the MCST shifts the cell membrane to close the potassium channel, thus resulting in hyperpolarization. Therefore, depolarization resulted in more pronounced nystagmus, which increased the sensitivity of the test.

Patients presenting with a history and physical examination of vestibular weakness, and a normal MWST result (<25% IAD) should complete the caloric test with cold irrigation, as the MCT is less sensitive for subgroups with less severe disease [7,19]. A diagnosis with UVW is valid if IAD in MWST is equal to or higher than 25% by using the MCA-2 formula, as it showed higher sensitivity and specificity (80% and 91%, respectively). Furthermore, the false PPV was 16.9% using the MCA-2 formula in MWST, lower than the reported studies of 48–78% [9,10] and the false NPV was 10.8% using the MCA-2 formula in MWST, lower than the reported studies of 29% [7,8]. This indicates that MCT results are a valid screening test in patients without HSN and with warm stimulation using the MCA-2 formula.

HSN is a failure factor that decreases the sensitivity and specificity of MCT [11,12,18,19,20,21]. A positive HSN of >4°/s in the VNG is considered an indication of BCT [5,6,13]. Other studies found the presence of HSN regardless of its velocity, which is an indication of BCT [11,18,19]. We studied 34 patients with HSN (mean, 2.25°/s) to analyze their effects on the final results. The calculations were performed with and without HSN adjustment. We found that the sensitivity increased using the MCA-2 formula and after HSN adjustment. Furthermore, the HSN affects the MCA results because the specificity was lower than expected in all formulas; we conclude that all patients with HSN and with an IAD < 25% after adjustment should complete the caloric test with cold irrigation to perform the BCT.

**Figure 4:**
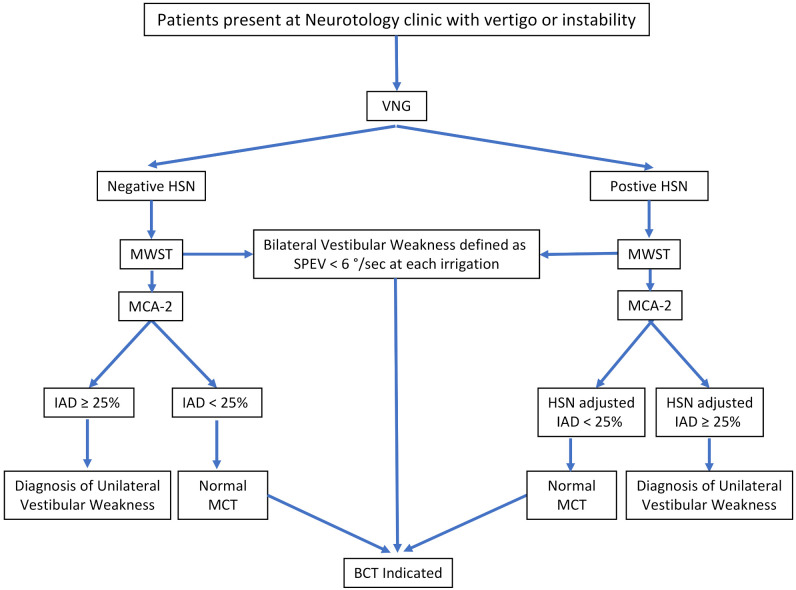
Diagnostic Algorithm of monothermal warm stimulating test. VNG: Videonystagmogram; HSN: Horizontal Spontaneous Nystagmus; MWST: Monothermal Warm Stimulation test; MCA: Monothermal caloric asymmetry; MCA-2 formula: MCA-2 = (Right − Left) × 100/(Right + Left); IAD: Inter-auricular Difference; SPEV: Slow-Phase Eye Velocity; BCT: Bithermal Caloric Test; MCT: Monothermal caloric test.

In some patients, the caloric responses in both ears were very weak or absent. When each of the four caloric irrigations had a peak slow component velocity lower than 6°/s, bilateral weakness was present [14]. The diagnostic algorithm for MWST is shown in Figure 4.

We studied the correlation between the two formulas used to measure MCA in comparison with BCT results. The Kappa agreements between the three formulas indicate moderate agreement, which is statistically acceptable. The MCA-1 formula is faster to apply than MCA-2 for those who calculate the IAD manually, but the MCA-2 formula showed better sensitivity, specificity, PPV, NPV, and ROC curve in MWST. This eventually makes the MCA-2 formula better for calculating the MCT.

### Limitation of the Caloric Test

Depending on the clinical and physical examinations, other vestibular and otolithic diagnostic tests can be performed to evaluate dizzy or vertiginous patients. The caloric test is non-physiological as it stimulates the lateral semicircular canal at 0.003 Hz with angular movements. The caloric test, both bithermal and monothermal, assesses only the function of the lateral semicircular canal; it will not provide information about the function of the vertical canals, saccule, or utricle. Furthermore, symptoms of UVW originating from the inferior vestibular nerve will not be detected by caloric testing. These patients need a video head impulse test (vHIT) that will test each canal separately and vestibular evoked myogenic potentials (VEMP) to assess the integrity of the saccule and utricle [2,22]. In addition, monothermal caloric test is unable to identify the directional preponderance of caloric nystagmus.

## 5. Conclusions

MCT is efficient as a screening test if the warm stimulus is used with the MCA-2 formula fixed at 25%. If present, HSNs should be adjusted. Negative IAD (normal) in the absence or presence of adjusted HSN or SPEV equal to or less than 6°/s at each right and left warm stimulation should be accomplished by the BCT. The vestibular work-up completed with other vestibular tests, such as the vHIT and VEMP tests, is recommended as it will assess the vestibular system as one unit and help in offering the patients an appropriate diagnosis.

## Figures and Tables

**Table 1:** Descriptive analysis for baseline patients’ demographic.

Patients’ Demographics N = 259		Healthy Controls N = 153 (59.1%)	Unilateral Vestibular Weakness (UVW) N = 106 (40.9%)	*p*-Value
Age (years) *	Mean ± SD	47.8 ± 14.1	53.8 ± 13.1	0.001
Gender	Females	92 (60.1%)	70 (66.0%)	0.404
	Males	61 (39.9%)	36 (34.0%)	

* Data are presented as mean ± SD and frequency (n [%]). SD: standard deviation

**Table 2:** Results of MCT for both warm and cold stimulation in comparison with BCT among non-HSN patients.

	Caloric Weakness Cutoff Points		MCT	Sensitivity	Specificity	Positive Predictive Value	Negative Predictive Value
Patients without HSN (N = 225)	BCA (20%)	MCA-1 (25%)	MWST	91.2% (85.1–97.4)	58.6% (50.6–66.6)	54.9% (46.4–63.3)	92.4% (87–97.8)
			MCST	85% (77.2–92.8)	67.6% (60–75.2)	59.1% (50.1–68.1)	89.1% (83.3–94.9)
		MCA-2 (25%)	MWST	80% (71.2–88.8)	91% (86.4–95.7)	83.1% (74.7–91.5)	89.2% (84.2–94.2)
			MCST	58.8% (48–69.5)	93.1% (89–97.2)	82.5% (72.6–92.3)	80.4% (74.3–86.4)

BCT: Bithermal caloric test, BCA: Bithermal caloric asymmetry, HSN: Horizontal spontaneous nystagmus, MCA: Monothermal caloric asymmetry, MCT: Monothermal caloric test, MWST: Monothermal warm stimulating test, MCST: Monothermal cold stimulating test.

**Table 3:** Evaluation of the screening effect of MCT for both warm and cold stimulation compared to the BCT in patients with horizontal spontaneous nystagmus (HSN) with and without HSN adjustments using the MCA-2 formula.

HSN Patients N = 34	Caloric Weakness Cutoff Points		MCT	Sensitivity	Specificity	Positive Predictive Value	Negative Predictive Value
**Without HSN adjustment**	BCA (20%)	MCA-2 (25%)	MWST	61.5% (42.8–80.2)	75% (45–105)	88.9% (74.4–103.4)	37.5% (13.8–61.2)
			MCST	73.1% (56–90.1)	62.5% (29–96)	86.4% (72–100.7)	41.7% (13.8–69.6)
**With HSN adjustment**	BCA (20%)	MCA-2 (25%)	MWST	81% (64.2–97.7)	53.8% (26.7–80.9)	73.9% (56–91.9)	63.6% (35.2–92.1)
			MCST	76.2% (58–94.4)	69.2% (44.1–94.3)	80% (62.5–97.5)	64.3% (39.2–89.4)

BCA: Bithermal caloric asymmetry, MCA: Monothermal caloric asymmetry, MCT: Monothermal caloric test, MWST: Monothermal warm stimulating test, MCST: Monothermal cold stimulating test.

## Data Availability

Data available on request due to restrictions (ethical).
